# Simultaneous occurrence of two B-cell malignancies: A case report

**DOI:** 10.3892/ol.2014.2185

**Published:** 2014-05-27

**Authors:** SHUJUAN ZHOU, YONGYONG MA, LAIXI BI, ZHIJIAN SHEN, KANG YU

**Affiliations:** Department of Hematology, The First Affiliated Hospital of Wenzhou Medical University, Wenzhou, Zhejiang 325000, P.R. China

**Keywords:** double B-cell malignancies, multiple myeloma, diffuse large B-cell lymphoma, DCEP regimen

## Abstract

The present study describes the case of a 75-year-old male with coexisting multiple myeloma and diffuse large B-cell lymphoma. Although the two malignancies are mature B-cell neoplasms, their clinical manifestations are very different and the clinical approaches used to treat these two types of tumor vary. The patient in the present case was diagnosed with the simultaneous existence of two different B-cell tumors and was successfully treated using the DCEP regimen. The simultaneous presentation of two mature B-cell neoplasms, types of hematological malignancy, is very rare, thus the present case is considered to be interesting.

## Introduction

Multiple myeloma (MM) and diffuse large B-cell lymphoma (DLBCL) are neoplastic processes that involve mature B cells. The reported instances of MM and DLBCL occurring in a single patient are extremely rare and the mechanism of pathophysiology remains poorly understood. Patients with MM have been reported to have a higher risk of developing secondary hematological malignancies (1–3). Although the two malignancies are derived from mature B cells, the clinical approaches used to treat these two types of tumor are different. The present study describes a case of coexisting MM and DCBL that was successfully treated using the DCEP regimen. Patient provided written informed consent.

## Case report

A 75-year-old male presented to the First Affiliated Hospital of Wenzhou Medical University (Wenzhou, Zhejiang) with DLBCL and was treated with chemotherapy in June 2009. Twenty days prior to admission, the patient suffered from abdominal distention and computed tomography (CT) revealed an abnormality of the ascending colon. The patient underwent a right hemicolectomy. Histopathologic examination and paraffin histology revealed a dense diffuse infiltration by large lymphoid cells. Further immunohistochemical analyses revealed positive labeling for the B-cell antigen, cluster of differentiation (CD) 20, CD79a, CD10, B-cell lymphoma (BCL)-6, melanoma associated antigen (mutated)-1, Epstein-Barr virus-encoded small RNA and the Epstein-Barr virus. Furthermore, a high proliferation index was detected using Ki-67 staining, which was 80%. The tumor cells were observed to be negative for CD138 and CD38. These findings led to the diagnosis of DLBCL. The patient presented with no fever or bone pain, however, exhibited weight loss. During the previous three months, the patient had lost 5 kg. The patient received further treatment at The First Affiliated Hospital of Wenzhou Medical University (Wenzhou, China). Upon physical examination, the patient appeared healthy, except for the surgical scar. Complete blood cell counts were as follows: Leukocytes, 3.4×10^9^/l; hemoglobin, 9.1 g/dl; and platelets, 156×10^9^/l. Blood analyses revealed the following: Serum β-2 microglobulin, 6.5 g/l; serum immunoglobulin (Ig)G, 31.9 g/l (reference interval, 7.51–15.6 g/l); serum IgA, 0.41 g/l (reference interval, 0.82–4.53 g/l); and serum IgM, 0.23 g/l (reference interval, 0.46–3.04 g/l). Immunofixation electrophoresis revealed the presence of monoclonal gammopathy (IgGλ). Bone marrow plasma cells were found to comprise 40% of the nucleated cells. Plain radiographic examination of the whole body did not demonstrate any abnormalities. However, emission (E) CT showed abnormal radioactivity in thoracic vertebrae T8–T10 and lumbar vertebra L2. Based on these findings, the patient was diagnosed with MM and DLBCL.

The patient received six cycles of DCEP chemotherapy, which consisted of: 20 mg Dexamethasone intravenously (IV), 0.4 g cyclophosphamide IV, 0.1 g etoposide IV and 15 mg cisplatin IV all on days 1–4. Following treatment, the IgG levels decreased to within the normal range, the bone marrow was normal and immunofixation electrophoresis revealed no monoclonal gammopathy. Based on these findings, the treatment was terminated; however, the patient failed to attend the follow-up appointments in the outpatient department over the next two years. After three years, on July 2012, the patient was hospitalized again due to fatigue, numbness and weakness of the limbs, as well as dysarthrosis of the right leg. The complete blood cell counts were as follows: Leukocytes, 1.4×10^9^/l; hemoglobin, 5.9 g/dl; and platelets, 15×10^9^/l. Blood analyses revealed the following: Serum β-2 microglobulin, 7.07 mg/l; serum IgG, 19.1 g/l; serum IgA, 0.27 g/l; serum IgM, 0.22 g/dl; and serum lactate dehydrogenase, 208 U/l. Immunofixation electrophoresis revealed a persistent IgGλ monoclonal gammopathy. Bone marrow plasma cells comprised 27.8% of the nucleated cells. A whole body positron emission tomography (PET)-CT scan was performed. Multiple lesions showing 2-fluoro-2-deoxy-d-glucose (FDP) uptake were observed in the first lumbosacral vertebral body, as well as in the lymph nodes in the neck and mediastinum.

The patient received one cycle of ECHOP chemotherapy (0.1 g etoposide, 1.0 g cyclophosphamide IV, 80 mg epirubicin IV and 2 mg vincristine IV all on day 1, and 75 mg prednisolone *per os* on days 1–5). However, the patient suffered from serious bone marrow suppression and an abdomen infection, and despite supportive care, the patient succumbed due to the infection.

## Discussion

In the present study, upon detection of a paraprotein in the patient, the most likely diagnosis was considered to be a lymphoma. Paraproteins have previously been reported in patients with lymphoma (4–5); however, this diagnosis was excluded based on subsequent examinations, including peripheral blood count, and bone marrow and skeletal system examinations. The patient met the criteria for the diagnosis of MM. MM is a neoplastic process that involves the B-cell lineage, which manifests as a plasma cell proliferative disorder in the bone marrow, monoclonal protein in the blood and/or urine, and ultimately results in organ damage, including bone destruction, anemia and renal failure. In the present study, the patient’s bone marrow plasma cell distribution was 40% and the presence of serum monoclonal protein was confirmed via immunofixation electrophoresis, thus, the patient was diagnosed with MM. Furthermore, bone lesions were identified in the vertebrae, which was confirmed using PET-CT and ECT scans. The patient was found to have symptomatic plasma cell myeloma with anemia and bone lesions and therefore, required therapy.

DLBCL is the most common type of B-cell lymphoid neoplasm and it is derived from B cells in the lymph node germinal center that have undergone somatic mutation in the Ig genes. DLBCL is a heterogeneous group of aggressive lymphomas of large, transformed B cells that originates from B cells from different differentiation stages, including germinal center B cells and plasma cells, which are blocked from undergoing terminal plasma cell differentiation. The malignant cells have a high rate of proliferation and express mature B-cell markers including CD20 and CD79a, however, not CD138, which may be detected using immunohistochemical analysis. In the present case, the patient’s tumor cells exhibited positive labeling for the B-cell antigens CD20, CD79a, CD10 and BCL-6, as well as a high proliferation index, which was detected using Ki-67 staining and was found to be 80%. The tumor cells were observed to be negative for CD38 and CD138. Based on these findings, the patient was diagnosed with DLBCL of the colon.

MM and DLBCL cell lines originate from B cells. However, the two malignancies may be distinguished by analyzing their distinct Ig surface proteins. The simultaneous occurrence of MM and DLBCL in the present case requires various considerations.

MM and DLBCL may demonstrate distinct clonal proliferation, although there are certain proposed risk factors that are shared between MM and non-Hodgkin lymphoma (NHL). Different types of cancer share numerous etiologic factors and the pertinent genetic traits may have low to moderate penetrance, and be driven by a number of gene-environment and gene-gene interactions (6). For example, previous studies have shown that exposure to ionizing radiation increases the risk of developing MM and NHL, as well as solid tumors (7–10). Furthermore, previous studies have indicated that exposure to chlorinated solvents is associated with the development of NHL and MM (11,12). Patients with MM have also been reported to exhibit a higher risk of developing secondary hematological malignancies (1–3). This predisposition may be aggravated by the immunodeficiency that is associated with MM. Various MM-associated tumors have also been reported (1,13). Thus, a number of environmental and behavioral risk factors, combined with host susceptible genetics, may lead to a secondary malignancy in a patient.

In the present case, the two malignancies may have been different manifestations of a unique clonal disorder. It has been proposed that NHL clones continue to mature and give rise to MM (14), malignant lymphoma disguised as MM (15) or the spread of cancerous cells from a single clone to multiple sites. If that were the case, MM and NHL may share the same Ig gene rearrangements. However, in the present study, IgH rearrangements were not performed, therefore, this hypothesis cannot be confirmed.

The specific mechanisms underlying the simultaneous presentation of two B-cell malignancies have yet to be elucidated; therefore, there are no uniform treatment guidelines. With the lack of guidelines for the management of such diseases, the treatment modality should be determined based on the doctor’s experience, the characteristics of the patient’s illness and the performance status.

At present, the first-line therapy for DLBCL is R-CHOP. Second-line therapies include CEPP, MINE and CEOP. The National Comprehensive Cancer Network panel includes a bortezomib, lenalidomide, melphalan and prednisone regimen as a Category 1 option for the primary treatment of patients with MM who have not received a transplant (16). Salvage therapy includes DCEP. In the present study, the patient was 75-years-old and was not a candidate for transplant or high-dose therapy. The DCEP regimen is similar to the second-line therapy, which is used to treat DLBCL; therefore, in the present case, the DCEP regimen served as a simultaneous treatment for MM and DLBCL. The patient achieved complete remission from MM. However, as a PET-CT scan was not performed following six cycles of DCEP, whether the patient achieved remission from DLBCL remains unknown. Although, the PET-CT scan that was performed following relapse revealed no FDP uptake in the primary site of disease in abdomen.

In conclusion, the present study describes the case of a patient with two separate B-cell malignancies and has shown that the DCEP regimen may be administered to treat patients exhibiting DLBCL and MM simultaneously. Furthermore, the present case demonstrates that the presence of paraproteins in patients with DLBCL may not be associated with the lymphoma.
